# Linking high parity and maternal and child mortality: what is the impact of lower health services coverage among higher order births?

**DOI:** 10.1186/1471-2458-13-S3-S7

**Published:** 2013-09-17

**Authors:** Emily Sonneveldt, Willyanne DeCormier Plosky, John Stover

**Affiliations:** 1 Futures Institute, Glastonbury, CT, USA

## Abstract

**Background:**

A number of data sets show that high parity births are associated with higher child mortality than low parity births. The reasons for this relationship are not clear. In this paper we investigate whether high parity is associated with lower coverage of key health interventions that might lead to increased mortality.

**Methods:**

We used DHS data from 10 high fertility countries to examine the relationship between parity and coverage for 8 child health intervention and 9 maternal health interventions. We also used the LiST model to estimate the effect on maternal and child mortality of the lower coverage associated with high parity births.

**Results:**

Our results show a significant relationship between coverage of maternal and child health services and birth order, even when controlling for poverty. The association between coverage and parity for maternal health interventions was more consistently significant across countries all countries, while for child health interventions there were fewer overall significant relationships and more variation both between and within countries. The differences in coverage between children of parity 3 and those of parity 6 are large enough to account for a 12% difference in the under-five mortality rate and a 22% difference in maternal mortality ratio in the countries studied.

**Conclusions:**

This study shows that coverage of key health interventions is lower for high parity children and the pattern is consistent across countries. This could be a partial explanation for the higher mortality rates associated with high parity. Actions to address this gap could help reduce the higher mortality experienced by high parity birth.

## Background

The link between high parity and maternal and child mortality is well documented in existing literature and commonly supported among reproductive health experts [1-3]. This relationship can be seen at the international level, with countries with higher total fertility rates having higher maternal and child mortality (such as Nigeria and Mali). , as well as at the national level (variation between states in India), with analysis showing mortality rates increase as parity increases. However, the mechanism that this relationship works through is still unknown.

There have been various theories, ranging from biological factors concerning the woman to sociological factors regarding access and consumption of health services. The most familiar of these posits that the physical and caloric demands of repeated pregnancy, in combination with the physical and caloric stresses of life at a subsistence level, result in a “maternal depletion syndrome” [4-6]. In the fifty years since the origin of this theory, debate continues as to whether parity is positively associated with outcomes such as anemia, low maternal weight gain, and low-birth weight, given confounding factors such as maternal age, the health status of mothers that are able to achieve high parity, breastfeeding duration, the spacing between pregnancies, changes in body fat distributions through successive pregnancies, and rapidly shifting diets across the globe [7-14]. A second theory focuses principally on poorer nutritional status of high parity children as a result of lesser parental investment and/or competition between siblings for finite resources [15-17]. However, proving these correlations is complicated by the need to account for household composition such as the child-dependency ratio within the household, the age and gender of the person heading the household, and the presence of twins or triplets. Further, sex preference, whether the children were from mistimed or unwanted pregnancies, and cultural differences regarding the support of weaker children also seem to play a role [18-21].

This analysis seeks to build upon the discussion of parental investment, by looking at health care decision making in regard to utilization of maternal and child health interventions as parity increases. It could be assumed that high parity women are overall less likely to access health services for themselves or their children, due to a strain on resources, the inability to find time given the need to care for so many children, or a reduced sense of urgency as pregnancies and childhood illnesses become repetitive. It could also be that lack of physical access to health facilities might be a barrier to accessing family planning services which could lead to more higher parity births. Using DHS data, this analysis will test the hypothesis that higher parity leads to increased maternal and child mortality because higher order births are less likely to receive critical maternal and child health interventions compared to lower order births.

## Methods

The analysis was completed in two stages. The first stage analysed differences in the health service coverage levels by parity and the second used the LiST model to link these differences to changes in mortality.

Country level demographic and health survey (DHS) datasets were used to complete the first part of the analysis because they contain the necessary information on coverage for both maternal and child health services. DHS data within the last 5 years were available for ten high-parity countries, representing Africa (both Francophone and Anglophone), Asia, and Latin America. These countries are Benin, Chad, Ghana, Malawi, Mali, Niger, Nigeria, Tanzania, Haiti, and Pakistan. Sample sizes for these countries are large with an average of 41,000 total births per country. The average number of births declines by parity from 9700 for parity one but is still large at 830 at parity 10+.

Nine maternal health interventions and eleven child health interventions were available for this analysis (Table 1). For all interventions other than institutional delivery and skilled birth attendance, the structure of the DHS survey questionnaire prevents analysis on births other than the most recent birth.

**Table 1:** Maternal and child health interventions included in the analysis

Maternal Interventions	Child Interventions
4+ ANC Visits	Vaccines: measles, DPT3, polio 3, BCG
< 4 months pregnant at first visit	Treatment sought from a health provider for diarrhea
Took any iron supplementation	ORS taken for diarrhea
Took any non-traditional antimalarial	Treatment sought from a health provider for cough and shortness of breath
2 or more doses of TT	Treatment sought for fever
Institutional delivery	Took any non-traditional antimalarial
Skilled birth attendance (doctor or nurse/midwife only)	Given nothing other than breastmilk in the first 3 days
PNC for those that delivery outside of a health facility	Child lives with respondent
PP Vitamin A supplementation	

Several interventions that were on the original list to draw from the DHS datasets were subsequently dropped because data were not available for more than a few countries, or the necessary coding was too individual by country to allow for direct comparison across countries. These interventions included: folic acid supplementation, clean cord care practices and essential newborn care, exclusive breastfeeding 0-6 months, appropriate complementary feeding 6-24 months, Hib vaccine, pneumococcal vaccine, vitamin A supplementation in children, zinc supplementation, antibiotics for dysentery, antibiotics for pneumonia, and therapeutic feeding.

The DHS birth data set for each of the countries was used to categorize births by birth order. Birth orders one through nine were analysed individually and the remaining were collapsed into a ten plus category. These data were used to examine coverage of health services as a function of the parity of the child. Chi square tests were used to identify which of the maternal health interventions had significant relationships between coverage and parity.

Many studies have shown disparities in maternal deaths by poverty status [22]. Therefore, we also controlled for poverty to see if that affected the relationship between coverage and parity. This analysis was done with linear regression using birth order as the dependent variable and the intervention plus the quintile indicator in the DHS data set as independent variables. These analyses were performed using STATA9.

The second part of the analysis used the Lives Saved Tool (LiST) [23] to estimate the impact the differing levels of coverage have on maternal and child mortality. The LiST model disaggregates maternal and child mortality by cause of death and then estimates the effects of different interventions in reducing mortality from specific causes. Some interventions, such as vaccines, act directly to reduce mortality from a specific cause, such as measles, while others act indirectly through intervening factors such as the effects of nutrition on stunting or breastfeeding on diarrheal incidence. The model contains 70 interventions organized into seven categories: periconceptual, pregnancy, childbirth, breastfeeding, preventive, vaccines, and curative.

The LiST model was used to create multiple scenarios that allow information on coverage to be compared to child and maternal mortality in various formats. This includes: (1) the isolated mortality impact of changes in coverage by parity among the individual interventions described above, (2) the full potential mortality impact from all the interventions described above, and (3) the full potential mortality impact of coverage of all interventions included in the LiST model.

The first scenario, *Intervention-specific Effects*, was used to estimate the contribution of lower coverage for individual interventions in high parity women to maternal and child mortality. This was done using only the significant interventions from the first part of the analysis. Health service coverage for the third birth for each intervention was used to reflect coverage for low parity women and coverage for the sixth birth was used to reflect coverage for high parity women. The significant interventions were scaled up by the point difference in coverage between the 3rd and 6th births (all other interventions were held constant). For example, in Niger the increase in coverage for institutional delivery from the sixth birth order to the third was 29.21 percentage points. Therefore, coverage was changed from its current value of 18.0% to 47.2%. The reductions in mortality due to scaled up health service coverage illustrate the link between our significant interventions and maternal and child mortality.

We also created additional LiST scenarios to provide a context to understand the real potential impact on mortality and to estimate the overall potential reduction in U5MR and MMR due to changes in coverage. Second and third LiST scenarios were created to provide this comparison. The second scenario, *Combined Intervention Effects*, scaled up coverage of all significant interventions to 99% in order to illustrate the full potential of these interventions to affect mortality. The third scenario, *Maximum Effects*, scaled up coverage of all interventions in LiST to 99% in order to illustrate the proportion of the total reduction in mortality that might be possible that could be due to just those interventions where parity may play a role in limiting coverage.

## Results

### Maternal health coverage

**Figure 1:**
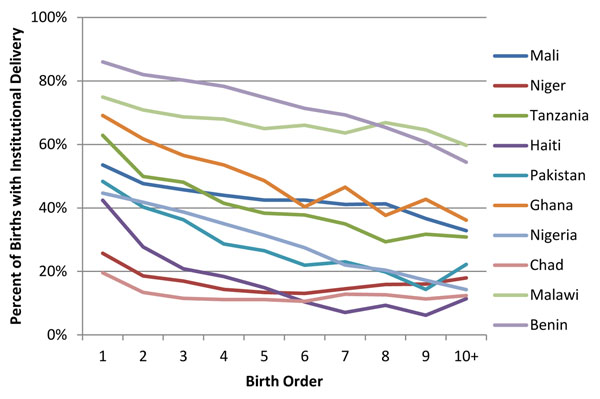
Coverage of institutional delivery by birth order

**Figure 2:**
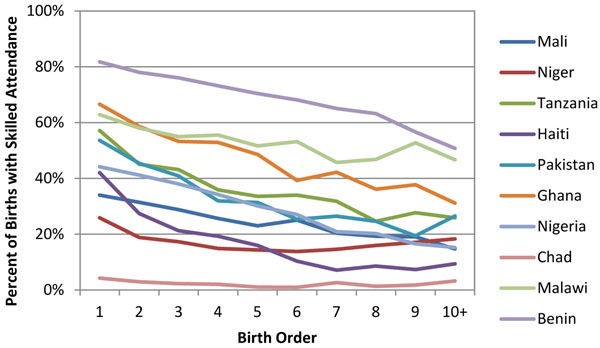
Coverage of skilled birth attendance at delivery by birth order

Overall, there is a trend toward lower utilization of maternal health services as birth order increases. Figures 1 and 2 show the two indicators, institutional delivery and skilled birth attendance, with the clearest linear relationship. For all countries more first order births are likely to occur in a health facility and/or by a skilled attendant than birth order ten plus, with the largest differences seen in Ghana and smallest in Chad.

For the majority of the maternal health interventions there is a significant relationship between the intervention and parity. Table 2 below highlights the indicators that are significant at the .05 level by the chi-square test for each intervention by country (shaded color denotes significance). With the exception of post-natal check-ups, all of the interventions are significant in the majority of countries and two of the interventions, institutional delivery and skilled birth attendance, are significant across all countries.

**Table 2:** Maternal health interventions that are significantly associated with parity by country

	ANC Care	Months Pregnant at First ANC visit	Iron Supplementation	Took Antimalarials	Tetanus Toxoid Before Birth	Institutional Delivery	Skilled Birth Attendance	Post-Natal Check-Up	Vitamin A Supplementation Post-Partum
Mali	.**00**	**.00**	.**01**	.08	**.03**	**.00**	**.00**	.43	**.00**
Niger	.25	**.01**	.30	**.00**	**.00**	**.00**	**.00**	.39	**.04**
Tanzania	**.00**	**.01**	**.01**	**.00**	**.00**	**.00**	**.00**	.01	**.00**
Haiti	**.00**			.23	.11	**.00**	**.00**	.14	**.00**
Pakistan	**.00**	**.00**	**.00**	**.00**	**.00**	**.00**	**.00**	.27	**.00**
Ghana	**.00**	**.00**	.56	.18	.01	**.00**	**.00**	.66	.43
Nigeria	**.00**	**.00**	**.00**	**.00**	**.00**	**.00**	**.00**	**.00**	**.00**
Chad	.12	.07	.10	.75	.55	**.00**	**.00**	.79	
Malawi	**.014**	**.05**	**.00**	**.00**	**.00**	**.00**	**.00**	.65	.76
Benin	**.00**	**.00**	**.00**	**.00**	**.00**	**.00**	**.00**	.14	.10

Values in each cell represent the p-values from a chi-square test. Bold numbers have p-values less than 0.05.

### Child health coverage

**Figure 3:**
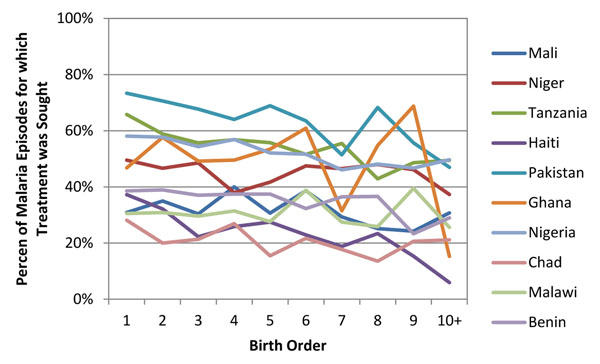
Percent that sought treatment for child’s malaria by birth order

**Figure 4:**
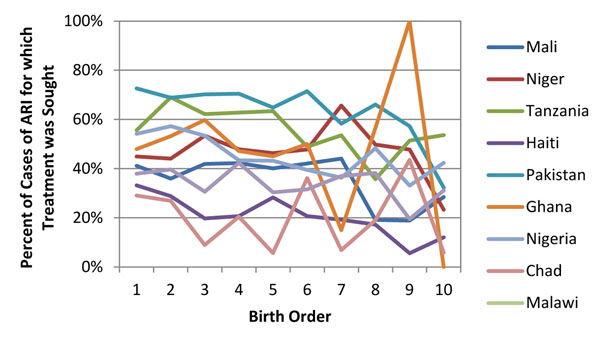
Percent that sought treatment for child’s acute respiratory infection

We found that child health indicators also show a relationship between coverage and parity but the relationship is not as strong linearly as seen in the maternal health interventions. Figure 3 shows one of the more linear relationships with child health interventions, treatment sought for malaria while Figure 4 shows one of the less linear relationships with treatment sought for acute respiratory infections. In both examples there is higher coverage at parity one compared to parity ten plus, but there is not a pure linear decline in coverage as birth order increases.

There were also fewer significant relationships between intervention coverage and parity among the child health interventions. Chi square tests found that five of the eleven indicators are significant in a majority of the countries, with breastfeeding and child’s residence having a significant relationship with coverage in most countries. Table 3 below highlights the indicators that are significant at the .05 level for each intervention by country (shaded color denotes significance). Treatment sought and treatment given for diarrhea are two of the interventions that are the least significant, with each only significantly related to coverage in two countries.

**Table 3:** Child health interventions that are significantly associated with parity by country

	Measles Vaccine	DPT Vaccine	Polio Vaccine	BCG Vaccine	Tx Sought for Diarrhea	ORS Taken for Diarrhea	Tx Sought for ARI	Tx Sought for Fever (Malaria)	Any Antimalarial Taken for Fever	Child Given Nothing other than Breastmilk in First 3 Days of Life	Child <5 lives with Respondent
Mali	.49	.20	.28	.39	.86	.71	.25	.27	.52	**.02**	**.00**
Niger	.43	.07	.24	.07	.42	.12	.**01**	.32	.96	.55	**.00**
Tanzania	.**00**	.74	.**01**	.33	.62	**.01**	.17	**.02**	.42	**.00**	**.00**
Haiti	.41	.25	.33	.22	.12	**.00**	.01	**.01**	.12	**.01**	**.00**
Pakistan	**.01**	**.01**	.29	**.04**	**.03**	.33	.00	**.00**	**.05**	**.00**	.88
Ghana	.26	**.02**	**.03**	.06	.64	.90	.13	.29	**.00**	.89	**.00**
Nigeria	**.00**	**.00**	**.00**	**.00**	.35	.20	**.01**	**.01**	**.03**	**.00**	**.00**
Chad	.13	.27	.08	.29	.**03**	.06	.14	.16	.59	.79	.20
Malawi	**.01**	**.00**	**.01**	**.05**	.87	.99	.11	.72	.12	**.00**	**.00**
Benin	**.05**	**.00**	**.04**	**.00**	.75	.71	.10	.11	.33		**.00**

Values in each cell represent the p-values from a chi-square test. Bold numbers have p-values less than 0.05.

### Controlling for poverty for maternal health interventions

All of the maternal health indicators that were found to have a significant relationship with parity in the first stage of analysis (as shown in table 2) were re-examined after controlling for poverty. Table 4 shows the results from this analysis, with gray-shaded boxes showing the indicators that were excluded because they were not previously significant and white boxes showing indictors that were no longer significant after adding the control for poverty. The majority of maternal health indicators maintained their significant relationship with parity even when controlling for poverty. Although this does not mean that poverty is not related to changes in coverage for health services, it does suggest that controlling for poverty does not negate the previously found significant relationships.

**Table 4:** Maternal health interventions that are significantly associated with parity after controlling for poverty

	ANC Care	Months Pregnant at First ANC Visit	Iron Supplementation	Took Antimalarials	Tetanus Toxoid Before Birth	Insitutional Delivery	Skilled Birth Attendance	Post-Natal Check-Up	Vitamin A Supplementation Post-Partum
Mali	.11	**.00**	.34		.09	**.00**	**.00**		.42
Niger		**.01**		**.02**	**.00**	**.00**	**.00**		.39
Tanzania	**.00**	**.00**	**.00**	**.00**	**.00**	**.00**	**.00**	**.00**	**.00**
Haiti	**.00**	**.00**	.18			**.00**	**.00**		.05
Pakistan	**.00**	**.00**	.**00**		**.00**	**.00**	**.00**		.04
Ghana	.11	**.00**			**.01**	**.00**	**.01**		
Nigeria	**.00**	**.00**	**.00**	**.00**	**.00**	**.00**	**.00**	**.00**	**.00**
Chad						**.00**	**.00**		
Malawi	.27	.30	**.00**	.67	**.00**	**.00**	**.00**		
Benin	**.00**	**.00**	**.00**	**.00**	**.00**	**.00**	**.00**		

Values in each cell represent the p-values from a chi-square test. Shaded cells have p-values less than 0.05. Blank cells are those where the relationship was not significant before controlling for poverty.

### Using LiST to estimate changes in mortality

The second part of the analysis used the LiST model to estimate the effect of these differing levels in coverage by birth order on maternal and child mortality, using the Maternal Mortality Ratio (MMR) and under-five mortality rate (U5MR) for this measurement.

Analysis of this first scenario, *Intervention-specific Effects*, found there to be an average decrease in U5MR of 12% (range: 1%-29%) and an average decrease in the MMR of 22% (range: 2%-67%). The main reduction for the U5MR came from countries where there was greater health service coverage for the third birth for ORS and care for pneumonia, while greater coverage of institutional delivery and skilled birth attendance for the third birth order were important for reductions in the MMR.

The second scenario, *Combined Intervention Effects*, found that the change in coverage due to reducing parity from 6 to 3 accounts for 33% of the potential decrease in U5MR and 36% of the potential decrease in MMR that could be achieved by scaling up all of those interventions to 99%. This suggests that increasing health service coverage for high parity births to match that of low parity births for our identified significant interventions would account for about one third of the total potential mortality reduction achievable. The LiST model does not specify coverage by parity so we could not model this situation exactly but we approximated it by comparing mortality if all children had coverage similar to parity 6 coverage and if all children had coverage similar to parity 3 coverage.

The third scenario, *Maximum Effects*, found that the decrease in mortality associated with scaling up interventions with a significant coverage-parity correlation by the average difference in coverage between parity 6 and 3 accounts for 17% of the potential decrease in U5MR and 36% of the potential decrease in MMR that would be achieved by scaling up all interventions in LiST. This confirms that the significant interventions found in the first part of the analysis have a large impact on maternal mortality and that increasing coverage of high parity births could contribute to a large reduction in maternal mortality.

## Conclusions

This analysis contributes to the existing discussion and evidence base on the relationship between parity and mortality by identifying specific differences in health care decision making and utilization of health services between low and high parity births and the resulting relationship with both maternal and child mortality.

Our results showed a significant relationship between maternal and child health interventions and birth order, even when controlling for poverty. Maternal health interventions were more consistently significant across countries, while child health interventions showed fewer overall significant relationships and more variation both between and within countries. The differences in health seeking behaviour for both maternal and child health services found in this analysis show that higher order births are less likely to be covered by critical maternal and child health interventions than lower order births. Importantly, the maternal health interventions that are known to link directly to mortality (institutional delivery and delivery with a skilled birth attendant) showed the highest level of significance in all countries. For the child health interventions, some of the interventions that we know are linked to child mortality, such as vaccinations, were not significant in all countries. This could be due to the methodology used in this analysis. In countries with high overall coverage there might not be enough variation in coverage levels to show a significant relationship.

The reasons for these differences remain unclear. It is unknown if there is a desire to seek services but barriers related to cost, distance, or time prevent care from being received, or if there is a belief that caring for previous childhood illness for older children have prepared caregivers to respond to a childs illness without seeking medical attention.

Most of the relationships between parity and coverage were still significant after controlling for poverty. Although there are many aspects of poverty that are likely not reflected in the DHS quintile variable, and many may argue its ability to really measure poverty, it was used because it allows a standard metric to be applied across all countries. Ideally, coverage would be estimated by quintile, but sample size limits the ability to perform that analysis.

The second part of the analysis linked these changes in coverage to reductions in maternal and child mortality. Increasing coverage for high order births to be equivalent to coverage for low order births showed large decreases in both maternal and child mortality. However, as in the first part of the analysis, impact was greater on maternal death compared to child deaths. This is potentially due to differences in the contributing factors, periods of risk, and causes of maternal and child deaths. The time period that a woman has the highest elevated risk of maternal death is during delivery and immediately postpartum. If she is with a skilled health provider during this time period, her risk of death can be drastically reduced. This is supported by our findings that changes in coverage for two maternal health interventions, skilled birth attendance and institutional delivery, are the largest contributors to reducing maternal deaths. The child health interventions included in the analysis do not have a comparable overarching intervention that responds to a specific time of elevated risk, so a smaller reduction in child deaths is unsurprising.

The potential reduction in U5MR by scaling up just those interventions that have a significant parity-coverage relationship is smaller than the reduction estimated when all interventions are scaled up (17% compared to 33%). This suggests that our identification of significant interventions did not capture some important interventions. This is likely due to the data source. As explained earlier, many interventions were excluded from this analysis because they were not included in all of the countries datasets or country specific coding was too unique to allow for cross-country comparison. It is possible that redoing the analysis with different datasets that allow for an increase in the number of interventions analysed or repeating the analysis with only countries that have complete and/or comparable DHS datasets would show different results for the child health interventions.

This analysis suggests that differences in both maternal and child mortality by parity is at least partly due to lower coverage of health services among higher order births. Importantly, it suggests that efforts to increase coverage of a select set of interventions have the potential to decrease maternal mortality by a third and child mortality by about a quarter. However, this analysis also suggests that while differences in health seeking behaviour by birth order contribute to the differences in mortality, they do not explain all of the variation. Additional analysis will need to be done to identify the other factors that contribute towards higher rates of death for both women and children for high order births.

## Competing interests

We report no conflicts of interests.

## Authors' contributions

ES and WDK conducted the statistical analyses, ES and WDK prepared the manuscript, and JS contributed to the study design and revised the manuscript.

## Declarations

The publication costs for this supplement were funded by a grant from the Bill & Melinda Gates Foundation to the US Fund for UNICEF (grant 43386 to "Promote evidence-based decision making in designing maternal, neonatal, and child health interventions in low- and middle-income countries”). The Supplement Editor is the principal investigator and lead in the development of the Lives Saved Tool (LiST), supported by grant 43386. He declares that he has no competing interests.

This article has been published as part of *BMC Public Health* Volume 13 Supplement 3, 2013: The Lives Saved Tool in 2013: new capabilities and applications. The full contents of the supplement are available online at http://www.biomedcentral.com/bmcpublichealth/supplements/13/S3.
